# Multi-Omic Graph Diagnosis (MOGDx): a data integration tool to perform classification tasks for heterogeneous diseases

**DOI:** 10.1093/bioinformatics/btae523

**Published:** 2024-08-23

**Authors:** Barry Ryan, Riccardo E Marioni, T Ian Simpson

**Affiliations:** School of Informatics, University of Edinburgh, 10 Crichton Street, Edinburgh, EH8 9AB, United Kingdom; Centre for Genomic and Experimental Medicine, Institute of Genetics and Cancer, University of Edinburgh, Edinburgh, EH4 2XU, United Kingdom; School of Informatics, University of Edinburgh, 10 Crichton Street, Edinburgh, EH8 9AB, United Kingdom

## Abstract

**Motivation:**

Heterogeneity in human diseases presents challenges in diagnosis and treatments due to the broad range of manifestations and symptoms. With the rapid development of labelled multi-omic data, integrative machine learning methods have achieved breakthroughs in treatments by redefining these diseases at a more granular level. These approaches often have limitations in scalability, oversimplification, and handling of missing data.

**Results:**

In this study, we introduce Multi-Omic Graph Diagnosis (MOGDx), a flexible command line tool for the integration of multi-omic data to perform classification tasks for heterogeneous diseases. MOGDx has a network taxonomy. It fuses patient similarity networks, augments this integrated network with a reduced vector representation of genomic data and performs classification using a graph convolutional network. MOGDx was evaluated on three datasets from the cancer genome atlas for breast invasive carcinoma, kidney cancer, and low grade glioma. MOGDx demonstrated state-of-the-art performance and an ability to identify relevant multi-omic markers in each task. It integrated more genomic measures with greater patient coverage compared to other network integrative methods. Overall, MOGDx is a promising tool for integrating multi-omic data, classifying heterogeneous diseases, and aiding interpretation of genomic marker data.

**Availability and implementation:**

MOGDx source code is available from https://github.com/biomedicalinformaticsgroup/MOGDx.

## 1 Introduction

Heterogeneity in human diseases is a pertinent yet difficult issue that can confound the analysis of clinical trials, genetic association testing, drug responses, and intervention strategies. Heterogeneous diseases encompass any single disease with a broad range of manifestations or symptoms. Redefining such diseases through sub-type classification, symptomatic grading or similar has the potential to uncover new treatments, re-purpose old treatments or identify intervention strategies. This approach has already been shown to improve patient outcomes in a number of diseases (Brodlie *et al.* 2015). Performing classification tasks with heterogeneous diseases is a complex problem often requiring analysis of multiple types of data of varying scale and complexity, as such it needs analytic frameworks that are flexible and scalable.

The development of high-throughput sequencing technologies have made various types of biological data, coined ‘omic’ data, available. This increased availability has led to many novel bioinformatic analytical tools for individual omics. This individual omic focus has yielded positive outcomes, however, information from different omics are rarely compared. Identifying which is the most informative omic measure, and if the information captured in different omics overlap or are complementary, is largely unknown. Furthermore, identifying if omic and multi-omic analyses are fit for purpose, given the cost and time-consuming nature of omics, is an unmet need (Chen *et al.* 2023). Multi-omic analyses have shown success in improving early disease classification in Parkinson’s Disease, ALS and Alzheimer’s and as such are growing in popularity (Chen *et al.* 2023). We propose the selective integration of multiple informative omics can also increase the classification accuracy in heterogeneous diseases while meeting this requirement.

The use of a network taxonomy for multi-omic data integration has risen in popularity recently. Networks are easily integrated and can readily handle missing data. Gliozzo *et al.* (2020) and Li *et al.* (2015) show that representing data as a patient similarity network (PSN) can retain information and have superior or competitive performance compared to standard Euclidean methods for a single modality. netDx, developed by Pai *et al.* (2019), uses ridge regression and label propagation algorithms to integrate and perform ranked classifications on PSNs. Wang *et al.* (2021) define each modality as a single PSN, perform classification using a Graph Convolutional Network (GCN) and concatenate predictions into a cross-omic correlation tensor before making final label predictions. Li *et al.* (2022) perform classifications using a GCN on integrated PSNs. These methods are novel strategies for the integration of omic data, however, they have limitations in scalability, oversimplification, and handling of missing data. netDx’s integration of PSNs is analogous to majority voting, which has been found to be suboptimal for PSN integration (Gliozzo *et al.* 2022). MOGONET, by Wang *et al.* (2021), and MoGCN, by Li *et al.* (2022), cannot handle patients missing one or more omic measures, and can only handle a fixed number of modalities.

Hence, we introduce Multi-Omic Graph Diagnosis (MOGDx), a flexible tool for the integration of multi-omic data to perform classification tasks for heterogeneous diseases. MOGDx integrates modalities into a single PSN using the Similarity Network Fusion (SNF) algorithm, developed by Wang *et al.* (2014). MOGDx encodes the modalities using a Mulit-Modal Encoder (MME) and performs patient classification using a GCN. Backpropagation is performed jointly through the GCN and MME creating a fully supervised training pipeline, named Graph Convolutional Network with Multi-Modal Encoder (GCN-MME). The performance of MOGDx is benchmarked on the BRCA, Low-Grade Glioma (LGG) and Pan Kidney Cohort (KIPAN) datasets, with state-of-the-art performance demonstrated. MOGDx is the first tool of its kind which can handle missing patient data and has been specifically designed to integrate any number of data modalities or omic measures. We show the benefit of integrating multiple modalities and the importance of representing data as a PSN. We demonstrate that MOGDx can identify informative modalities as well as important omic markers relating to the targeted biomedical problem.

## 2 Results

### 2.1 Pipeline of MOGDx

**Figure 1.**
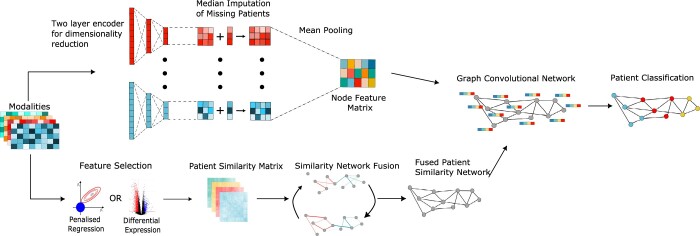
Pipeline of MOGDx–MOGDx takes any number of modalities as input. Feature extraction is performed to maximize similarities between patients. Individual PSNs are fused using SNF. The MME performs dimensionality reduction and imputation and as output provides a node feature matrix. Training is performed by back propagating through the GCN and MME for a fully supervised pipeline

We present MOGDx, a pipeline for the supervised classification of patients with heterogeneous diseases (Fig. 1). MOGDx takes as input any number of modalities. Due to the scale and availability of omic data such as genomic, transcriptomic, and proteomic datasets, it is common for early models such as MOGDx to take advantage of this. In this article, we will refer to data as modalities, despite only omic data being used. The reason for this is to highlight that MOGDx is not limited to omic data and could be used to integrate other modalities as they become more prevalent. The raw data are processed into matrices, with each row corresponding to a patient and each column a feature of that measure.

First, we calculate the similarity between patients in each modality. Depending on the modality (see Section 4), either differential expression or penalized regression is performed for feature selection. Patient similarity between these features is calculated using Pearson correlation where suitable, otherwise Euclidean distance. As is common practice, all patients are included in the network, with train, validation and test labels created during the training phase of the GCN-MME (Li *et al.* 2022). In this approach, features are discarded to reduce the complexity of the similarity calculation and to discourage uniform similarity scores for modalities such as DNAm which will have large number of redundant or similar features. A PSN is constructed from each modality’s similarity matrix using the K-Nearest Neighbours (KNN) algorithm. SNF is performed to fuse the PSNs into a single network. SNF is an algorithm which dampens weak relationships, enforces strong relationships and discovers novel relationships between patients. Crucially, it retains patient nodes if they are missing a sample from one or more modality (Wang *et al.* 2014).

The fused PSN and the modalities are input into the GCN-MME (Supplementary Fig. S1). Each modality is encoded using a two layer encoder. The first linear layer is of dimension D = 500, with the second layer being tuned to each modality by performing a hyperparameter search. Median imputation is performed on the second layer of each encoder to retain patients if they are missing from that modality. The second layer from each modality is then decompressed to a shared latent space using mean pooling, with its dimension tuned through a hyperparameter search also. This step encourages each modality to learn the same latent space and has been established in other similar encoder architectures (Yang *et al.* 2021). Including all features in the GCN-MME allows for interactions between features to be captured by the two layer encoder. Some features will be uninformative in a direct contrastive analysis, but the interaction between these features can also be informative. The shared latent space is the node feature matrix, required for training the GCN. Each row of the node feature matrix corresponds to a single patient node in the PSN. The node feature matrix and the PSN are combined and input into a GCN. The optimal MOGDx hyperparameter values per dataset are given in Supplementary Table S1.

A GCN architecture was implemented using the Deep Graph Library (version 1.1) in Python with a PyTorch backend. The GCN implemented consisted of two layers with intermediate relu activation and batch normalisation. The input dimension to the GCN is the same dimension as the node feature matrix. The output dimension of the second layer aligns with the number of classes for that classification task. The weights of both the GCN and MME are learned jointly by performing backpropagation through the entire GCN-MME. A GCN is a transductive algorithm requiring all nodes to be present during training and testing. During training, the loss scores of the validation and test nodes are removed from the calculation, thus preserving the integrity of the test and validation sets. This approach follows the semi-supervised training of GNNs outlined by Hamilton (2020) (see Section 4 for more detail). The MOGDx methodology integrates the predictive power of PSNs with a reduced representation of each modality’s characteristics. It obtains state-of-the-art predictive performance on the integration of network and node feature characteristics, exhibiting the benefit of including both. It is a command line tool for the supervised classification of heterogeneous diseases, which can be used for a wide range of biomedical and more applications.

### 2.2 Datasets

The performance of MOGDx is benchmarked on three different datasets from The Cancer Genome Atlas (TCGA); BRCA PAM50 sub-type classification, grade classification in LGG and KIPAN for kidney type classification. TCGA is an open source database of genomic, epigenomic, transcriptomic and proteomic data with over 20 000 primary cancer and matched normal samples spanning 33 cancer types. BRCA, LGG, and KIPAN are mature datasets from TCGA which have been implemented in similar research (Wang *et al.* 2021, Li *et al.* 2022). Data were downloaded using the TCGA Biolinks package (https://bioconductor.org/packages/TCGAbiolinks/).

All modalities available in the TCGA database were included, resulting in five types of omics data used for classification. The omic data types available are mRNA expression (mRNA) data, micro RNA expression (miRNA) data, DNA methylation (DNAm) data, Reverse Phase Protein Array (RPPA) data, and Copy Number Variation (CNV) data. All omics were processed using standard bioinformatic pipelines, where available. See Section 4 for further detail. All patients’ data were used irrespective if they were missing in some modalities, with specific details reported in Table 1.

**Table 1.** Sample availability per modality in TCGA datasets.

Dataset	Subtype	Patients per modality					Unique patients
		mRNA	miRNA	DNAm	RPPA	CNV	
BRCA	Luminal A	548	540	422	433	545	562
	Luminal B	204	141	141	177	205	209
	Basal	174	171	137	155	184	190
	HER2	81	76	46	75	80	82
	Normal-like	40	37	34	24	39	40

	Total	1047	1015	780	869	1053	1083

LGG	Grade 2	210	190	216	189	238	216
	Grade 3	227	218	241	200	212	241

	Total	437	408	457	389	450	457

KIPAN	KIRC	517	485	318	475	514	532
	KIRP	283	281	274	214	284	290
	KICH	46	27	66	63	66	66
	Total	846	793	658	752	864	888

BRCA PAM50 is a 50-gene signature used to sub-type breast cancer into five classifications; Normal-like, Basal-like, HER2-enriched, Luminal A, and Luminal B (Parker *et al.* 2009, Kensler *et al.* 2019). Patients included in this dataset have a mutation to their BRCA gene and therefore have a larger risk of developing breast cancer. Sub-typing by gene expression separates the carcinomas by varying biological properties and prognoses. For example, Luminal A has the best prognosis, while HER2 and Basal are considered more aggressive forms of cancer (Kensler *et al.* 2019). The KIPAN dataset consists of three categories separated by chromosomal differences (Tabibu *et al.* 2019). Clear Renal Cell Carcinoma (KIRC) is characterized by loss of chromosome 3p, Papillary Renal Cell Carcinoma (KIRP) is characterized by loss of chromosome 9p and Chromophobe Renal Cell Carcinoma (KICH) is characterized by loss of multiple other chromosomes. The LGG dataset consists of grade 2 and grade 3 which are characterized by the World Health Organisation based on their histopathologic characteristics (Forst *et al.* 2014). All of these datasets categorize a heterogeneous disease by a genetic association, making them suitable tasks for classification. They were chosen to demonstrate the generalizability of MOGDx to different diseases, as well as to benchmark the performance of MOGDx against other supervised network integrative methods (Wang *et al.* 2021, Li *et al.* 2022).

### 2.3 Performance and evaluation

The performance metrics used to compare the classification performance of MOGDx were accuracy and F1-score. The F1-score was calculated by the mean F1-score of each class, weighted by the size of that class. k-fold cross validation was performed with five randomly generated splits to obtain the mean and standard deviation metrics reported. Within each split, the training set was randomly split into training and validation sets to produce an overall train/validation/test split of 68%/12%/20%, respectively.

#### 2.3.1 MOGDx achieves improved F1-score and accuracy metrics compared to other supervised multi-omic network methods

The prediction accuracy and efficiency of MOGDx was compared to two other supervised multi-omic methods that used a network architecture and GNN, namely MOGONET by Wang *et al.* (2021) and MoGCN by Li *et al.* (2022). The three methods were tested on their ability to integrate DNAm, miRNA, and mRNA from the BRCA dataset of the TCGA. Although, MOGDx has the ability to include the union of patients, MoGCN and MOGONET do not, thus the three methods were subset to *n* = 698 patients who had a measurement in each of the three modalities. MOGDx outperforms both MOGONET and MoGCN when trained and tested on the same patients and dataset in both F1-score and accuracy. Computation time was calculated as the time taken to generate the networks, perform integration and train the graph neural network models. While MOGONET is more efficient, its accuracy is less than MoGCN and MOGDx. There is also an impact on efficiency when a larger number of patients are included.

The impact of imputation on MOGDx was tested by training on the union of patients (*n* = 1083) from the same three data modalities as before. As per Table 2, the accuracy decreases by approximately 4%, however, the number of patients included increases by 55.2%. Despite imputation, MOGDx’s accuracy remains greater than that achieved by MoGCN or MOGONET, although the F1-score is comparable to MoGCN. This illustrates the strength of MOGDx to retain patients while maintaining competitive accuracy.

**Table 2.** Comparison of multi-omic methods when integrating DNAm, miRNA and mRNA from BRCA dataset.

Dataset	Method	No. patients	Accuracy	F1-score	Computation time (min)
BRCA	MOGDx	698	0.917 ± 0.028	0.901 ± 0.033	20.7 ± 1.2
		1083	0.877 ± 0.013	0.858 ± 0.016	42.9 ± 3.6
	MOGONET	698	0.825 ± 0.006	0.816 ± 0.006	11.4 ± 0.9
	MoGCN	698	0.840 ± 0.024	0.851 ± 0.016	24.5 ± 1.2

#### 2.3.2 MOGDx demonstrates state-of-the-art performance when integrating a variable number of modalities despite including a larger number of patients

The overall performance of MOGDx was compared to metrics reported by similar supervised multi-omic integrative network methods as well as benchmark classification algorithms namely; Support Vector Machine (SVM), Elastic Net and gradient tree boosting (XGBoost). Table 3 shows MOGDx outperforms all benchmark classification algorithms on the BRCA and LGG datasets. This demonstrates the predictive power of integrating multiple modalities in these tasks. It achieves slightly poorer performance on the KIPAN dataset, however this mirrors the drop in accuracy due to imputation seen in Table 2 and indicates that a single modality is most predictive for this task.

**Table 3.** Summary of results.

Method	Dataset	No. modalities	No. patients	No. classes	Accuracy	F1-score
MOGDx	BRCA	4	1083	5	0.892 ± 0.008	0.873 ± 0.011
	BRCA	4	1043	4	0.904 ± 0.014	0.887 ± 0.016
	LGG	1	457	2	0.899 ± 0.016	0.881 ± 0.019
	KIPAN	4	888	3	0.958 ± 0.003	0.948 ± 0.004
MOGONET	BRCA	3	875	5	0.829 ± 0.018	0.825 ± 0.016
	LGG	3	510	2	0.816 ± 0.016	0.814 ± 0.014
	KIPAN	3	658	3	0.999 ± 0.002	0.999 ± 0.002
MoGCN	BRCA	3	511	4	0.898 ± 0.025	0.902 ± 0.024
	KIPAN	3	698	3	0.977 ± 0.017	0.977 ± 0.017
	BRCA	1	1047	5	0.856 ± 0.021	0.852 ± 0.023
SVM	LGG	1	457	2	0.681 ± 0.044	0.680 ± 0.044
	KIPAN	1	752	3	0.999 ± 0.003	0.999 ± 0.003
	BRCA	1	1047	5	0.809 ± 0.028	0.799 ± 0.033
Elastic Net	LGG	1	437	2	0.638 ± 0.047	0.637 ± 0.048
	KIPAN	1	752	3	0.993 ± 0.008	0.993 ± 0.007
	BRCA	1	1047	5	0.830 ± 0.020	0.815 ± 0.018
XGBoost	LGG	1	437	2	0.687 ± 0.023	0.686 ± 0.023
	KIPAN	1	752	3	0.985 ± 0.006	0.985 ± 0.006

The performance reported by MOGONET (Wang *et al.* 2021) was achieved using mRNA, miRNA and DNAm. The performance reported by MoGCN (Li *et al.* 2022) was achieved using CNV, mRNA and RPPA. These metrics were taken from their respective papers. The performances reported on SVM, Elastic Net and XGBoost methods were achieved using a single modality, from those available, which gave the highest accuracy.

MOGDx outperforms comparative integrative methods, MOGONET (Wang *et al.* 2021) and MoGCN (Li *et al.* 2022). In Table 2, we highlight the improved classification accuracy of MOGDx compared to MoGCN and MOGONET when the methods were tested on the same modalities and patients. Comparing the results of Tables 2 and 3, MOGDx outperforms its previous union integration model due to the inclusion of an additional modality, RPPA. Thus, on the BRCA dataset, MOGDx integrated mRNA, miRNA, DNAm, and RPPA for optimal performance. This highlights the benefit of flexible modality integration and demonstrates the ability of MOGDx to learn complimentary information from different modalities. It has poorer performance than its model which takes the intersection of patients but these differences can be accounted for by a 55.2% increase in the number of patients included and the imputation methods used. The Normal-like tumour subtype in the BRCA dataset is the most difficult classification subtype (shown in Supplementary Fig. S5). MoGCN exclude this subtype from their classification results as per Table 3 resulting in significantly greater performance compared to Table 2. The difficulty in classifying the Normal-like subtype is due to the small number of patients and the likelihood of these patients to go on to develop into one of the other sub-types. MOGDx achieves comparable performance to MoGCN despite including this additional subtype and more patients. This highlights its generalisability and its ability to classify on imbalanced datasets.

MOGDx identified a single modality, DNAm, which achieved optimal performance on the LGG dataset. All patients were available in this single modality as per Supplementary Fig. S2. While MOGDx did significantly outperform MOGONET on this dataset, there is a difference in the number of patients. MOGONET obtained their data from Broad GDAC Firehose which stores TCGA data version from 2016 which could explain this discrepancy. Finally, MOGDx achieves slightly lower accuracy and F1-score on the KIPAN dataset compared to those reported by MOGONET and MoGCN. It integrated miRNA, CNV, DNAm and RPPA for optimal performance. MOGDx, again, is able to include a larger number of patients due to its integration strategy. As previously, the lower performance of MOGDx in this dataset mirrors the degradation in performance seen in Table 2 when imputation methods are applied to include missing patients in one or more modality.

MOGDx incorporates greater number of patients and modalities in its methodology. MOGONET and MoGCN are limited to the intersection of patients, which reduce the number of patients included in their analysis when more modalities are included. This is evident in Supplementary Fig. S3 where there is a large variation and degradation in proportion of patients included when using an intersection of patients integration strategy. It can also be seen as SVM, Elastic Net, and XGBoost have greater number of patients available in the BRCA and KIPAN datasets, despite being trained on a single modality. Conversely, MOGDx uses a union of patients’ integration strategy, thus including all patients without a large degradation in performance. MoGCN is fixed to the integration of three modalities and albeit, in theory, MOGONET can be scaled for flexible integration, no such analyses have been performed. MOGDx has been specifically designed to allow any number of modalities to be included, which we show results in improved performance on the TCGA datasets as per Table 3 and Supplementary Fig. S4. This flexibility also provides insight into the information content of each modality. MOGDx, facilitates the analysis of individual as well as combined modalities. This can be utilised to identify the best combination of modalities and the added information content and cost effect of including additional modalities in an experiment.

#### 2.3.3 MOGDx can identify relevant biological markers of heterogeneous diseases

Ablation experiments were performed to determine which modalities were most predictive of each classification task, with the results shown in Supplementary Tables S3–S5. It was determined that mRNA and DNAm are highly predictive of BRCA and KIPAN, while only DNAm was predictive of LGG. mRNA and DNAm have established enrichment pipelines, thus these modalities were analysed for relevant biological markers in all datasets. Enrichment analysis was performed on the extracted features from the differential expression analysis on mRNA and penalised regression analysis on DNAm in all datasets. Table 4 shows the functional processes associated with mRNA and DNAm respectively for; BRCA PAM50 sub-type classification, KIPAN sub-type classification and LGG grade classification. Two gene sets were analysed, KEGG and MSigDB, for functional and biological enrichment processes and the full results of the enrichment analyses are given in Supplementary Tables S9 and S10.

**Table 4.** Summary of enriched mRNA and DNAm pathways in TCGA dataset.

Dataset	Modality	Gene set	
		KEGG 2021 Human	MSigDB Hallmark 2020
BRCA	mRNA	Pentose and glucuronate interconversions	G2-M Checkpoint
		Cell cycle	Androgen Response
	DNAm	Axon guidance	Estrogen Response Early
		Proteoglycans in cancer	Epithelial Mesenchymal Transition
LGG	mRNA	Nicotine addiction	G2-M Checkpoint
		Systemic lupus erythematosus	Epithelial Mesenchymal Transition
	DNAm	Axon guidance	Epithelial Mesenchymal Transition
		Proteoglycans in cancer	Estrogen Response Early
KIPAN	mRNA	Oxidative phosphorylation	Oxidative Phosphorylation
		Pentose and glucuronate interconversions	Epithelial Mesenchymal Transition
	DNAm	Axon guidance	Estrogen Response Early
		Human papillomavirus infection	Epithelial Mesenchymal Transition

The functional processes found to be enriched in the BRCA mRNA dataset in both gene sets are related to the life cycle of a cell, with good concordance between the two gene sets and corroborated with established research (Yarden *et al.* 2002, Deng 2006). Again, the DNAm enriched functional processes show good concordance between KEGG and MSigDB gene sets in BRCA. These enriched processes have established involvement with the development and growth of cancer tumours (Tzanakakis *et al.* 2020). The enriched DNAm processes in BRCA overlap significantly with LGG and KIPAN. This finding is unsurprising considering we have assessed three cancer tumour datasets. It does, however, highlight the ability of MOGDx to consistently identify relevant pathways, as well as motivating the use of DNAm to differentiate between different cancers and cancer subtypes. The concordance between enriched pathways in mRNA and DNAm differs more in BRCA than it does in LGG and KIPAN. This suggests that the enriched features of mRNA and DNAm are more orthogonal in BRCA, which could explain the inclusion of both of these modalities in the best performing model for this dataset. Conversely, in the LGG and KIPAN datasets, we see more overlap, with Epithelial Mesenchymal Transition enriched in both mRNA and DNAm. This indicates that similar information is captured in both these omics and thus, justifies the exclusion of one of them from the optimal modality combination. Given, mRNA was less predictive in both LGG and KIPAN, shown in Supplementary Tables S3 and S4, and the apparent overlap in information, it is less surprising that this modality was excluded from the optimal combination of modalities in these datasets.

## 3 Discussion

Disease heterogeneity has moved medical research from a population-based perspective towards a personalised approach where diagnosis, prognosis and treatments are selected based on biomedical characteristics. Driving this movement is the development of large, diverse omic technologies and studies which provide labelled biomedical data at unprecedented levels. The integration of these omic measures offer the opportunity to build quantitative models, which can aid the understanding of heterogeneous disease architectures and inform clinical guidance. Therefore, a tool which can flexibly incorporate omic measures and identify specific biomedical characteristics based on these labels has the ability to redefine heterogeneous diseases.

We propose MOGDx, a network integrative architecture for the classification of heterogeneous diseases. What separates MOGDx from its competitors is the flexibility in its integration of modalities, its inclusion of all available patient samples and its leverage of the predictive power of PSNs. MOGDx includes modalities, which either improves predictive performance or include patients who may only have samples in one modality. This allows users to fine tune to the most predictive modalities while incorporating the maximum number of patient samples in an analysis. In this analysis, we maximized data usage while demonstrating state-of-the-art performance on a variety of classification tasks. Fundamental to the predictive performance of MOGDx is the integration of PSNs. In this analysis, we have shown that patient similarity is a very effective determinant of heterogeneous disease sub-typing and grading. The use of PSNs is analogous to clinical diagnosis, where a diagnostician will compare a new patient to a database of similar cases. Similarly, MOGDx captures the variability in similarity and uses this to perform accurate sub-type classification and grading.

The GCN-MME is the most significant component of the MOGDx pipeline. It takes as input a fixed network and any number of modalities. The network is generated prior to training the GCN-MME model to reduce computation time and to aid generalizability of the GCN across the cross-validation splits. Generating a network for each modality and performing SNF during each cross-validated split would be computationally expensive and time intensive. Sensitivity of GCNs to a particular split in the data is a common pitfall during evaluation (Shchur *et al.* 2019). Having a fixed network and training the GCN in series with the MME forces the GCN-MME to learn more than favourable splits in the data and aids the generalisability of MOGDx.

The application of MOGDx has been benchmarked on three cancer datasets from the TCGA, namely; BRCA, LGG and KIPAN. Cancer is widely regarded as a highly heterogeneous disease; however, MOGDx was able to accurately classify breast cancer sub-types, kidney cancer sub-types and brain tumour grades from integrated omic data. MOGDx identified the optimal combination of modalities which resulted in greater patient coverage while maintaining a state-of-the-art classification performance compared to its competitors, as per Table 3.

Interpretability is an important aspect to consider for biomedical applications in order to transform research into novel diagnoses, grades or treatments. We have demonstrated the interpretability of MOGDx in several ways. Through leave one out experiments we have identified the modalities which are most predictive of the classification task, their most important features, and some enriched functional pathways. The use of different omics allows us to assay different parts of the biological systems involved in disease mechanism, their integration can help reduce biological noise improving signal and allowing for the identification of previously undetectable informative features. Understanding which omics are most predictive for a given disease can allow us to design more efficient and informative experiments, minimising impacts on patients and reducing costs. Further, because different omics modalities capture different components of the genetic and environmental contributions to disease, their integration can help us to gain a more complete picture of disease. We performed enrichment analysis on mRNA and DNAm modalities in all TCGA datasets. MOGDx was able to identify features enriched in processes and genes relating to the pathology and prognosis of the disease. These findings were supported by similar findings in the literature demonstrating MOGDx’s ability to identify important omic markers. They also helped explain why these modalities were included or excluded in the optimal integration. We have demonstrated in this work that the MOGDx architecture can successfully produce interpretable, explainable and reproducible insights into heterogeneous diseases. Moreover, the availability of large scale omic measures are a promising proving ground and avenue of research for integrative models such as MOGDx, however, its use can be easily extended to other modalities and applications as they become more prevalent.

Although commonly used, implementing GCNs results in limitations. It has been shown that transductive algorithms obtain higher accuracies than inductive, but they generalise more poorly (Lachaud *et al.* 2022). We aim to improve this generalisability by including cross validation splits within the GCN-MME architecture, however, this does introduce bias as the network is created using all patient data. This bias could explain why MOGONET achieves poorer accuracies compared to MOGDx and MoGCN as they extended their implementation of the GCN to the inductive setting. Inductive nodes both hide the labels and do not use the test data to update the embedding representations, thus aligning with a more classical supervised machine learning methodology. This also could explain why the accuracies obtained by MOGONET were more robust than MoGCN when training these methods on new datasets.

Transductive algorithms are also not tractable in a clinical setting. In a clinical setting, it will be required to perform predictions on new patients post model training and testing. For a transductive model, this would require recreation of the network and re-training of the model. Hence, it will be required to extend MOGDx to an inductive algorithm which does not require the entire network to be available during training and can make predictions on patients which are added to the dataset post model deployment. An emerging model of interest is the use of Graph Attention Network (GAT). GAT is an inductive algorithm which also offers another layer of interpretability and has shown promise in similar research (Bari Tanvir *et al.* 2024). In summary, MOGDx is a flexible and accurate classification tool which can be applied to a broad range of heterogeneous diseases.

## 4 Materials and methods

### 4.1 Framework of MOGDx

The framework for MOGDx incorporates four main components; (i) Pre-processing of modalities, (ii) Graph generation and SNF, (iii) GCN training and classification, and (iv) Interpretability of MOGDx.

### 4.2 Pre-processing of modalities

Pre-processing is performed to remove unwanted noise and variations in the data due to experimental or technical effects. Each modality was processed in a complimentary bioinformatics pipeline, if available. For example, mRNA and miRNA were processed using the standard DESeq2 pipeline outlined in Love *et al.* (2014). Briefly, genes which had either zero expression or zero variance in all samples were removed. Next, any samples which were more than 2 standard deviations from the mean node connectivity distance were removed. Differential expression was performed using a one-vs-the-rest methodology, and genes with an adjusted p-value below 0.05 were extracted.

The DNAm data downloaded from TCGA-Biolinks used multiple generations of Illumina Infinium DNA methylation arrays, where they have already been corrected and standardised using the SeSAMe (Zhou *et al.* 2018) pipeline. Further steps were taken to remove any CpG sites which contained missing values.

To overcome significant missingness in the CNV and RPPA datasets, sites which contained more than 50% missingness were removed, and mean imputation was performed. The CNV data was log transformed to give it a close to normal distribution. Informative features were extracted from DNAm, CNV and RPPA using penalised elastic net regression.

### 4.3 Graph generation and similarity network fusion

A patient similarity matrix is created for each modality. The Pearson correlation coefficient (Equation 1) between the extracted features was used as a measure of similarity where suitable, otherwise Euclidean distance (Equation 2). In modalities where correlation is not an appropriate measure, Euclidean distance is used.

(1) $$r=\frac{\sum \left(x_{i}-\bar{x}\right)(y_{i}-\bar{y})}{\sqrt{{\left(x_{i}-\bar{x}\right)}^{2}}{\left(y_{i}-\bar{y}\right)}^{2}}$$

(2) $$r(p,q)=\sqrt{\sum_{i=1}^{n}{\left(q_{i}-p_{i}\right)}^{2}}$$

The K-Nearest Neighbours (KNN) algorithm is used to build the graph with edges created between the 15 nearest neighbours. SNF (Wang *et al.* 2014) is applied to fuse the graphs into a single network representing the full spectrum of the underlying data. SNF allows complimentary information to be shared between modalities, and is effective in identifying novel relationships between patients. It also integrates missing patient samples inherently by complimenting a missing edge in one modality with the same relationship from others.

### 4.4 Graph convolutional network training and classification

GNNs are a powerful architecture for the learning of graph structure and information in a supervised setting. We implemented a GCN model from the Deep Graph Library in Python with a PyTorch backend. The differentiation between GCN and neural network architectures is their ability to learn from the local neighbourhood as opposed to handcrafted network features. The performance of GCN and other GNN architectures has been demonstrated on a variety of benchmark tasks, hence extending their application to a biomedical setting is an exciting avenue.

GCN requires two inputs. A network consisting of nodes and edges, and a vector of features for each node. For MOGDx, the network created was a PSN and the vector of features was a reduced feature representation from the MME. Formulating the GCN algorithm as a network represented by an adjacency matrix $A\in R^{n\times n}$ and a feature matrix $X\in R^{n\times d}$ where *n* is the number of patients and *d* is the latent dimension selected for the MME. The GCN then consists of stacked convolutional layers defined by Equation 3 (Kipf and Welling 2017).

(3) $$H^{l+l}=\sigma \left(LH^{\left(l\right)}W^{\left(l\right)}\right)$$

Where $L={\tilde{D}}^{-\frac{1}{2}}\tilde{A}{\tilde{D}}^{-\frac{1}{2}}$ is the normalized graph Laplacian; $\tilde{A}=A+I$ is the adjacency matrix; $\tilde{D}$ is the degree matrix of $\tilde{A}$; W is the weight matrix learned during training; σ is the non-linear activation function, ReLU activation in this case, $H^{\left(l\right)}$ is the input to each layer and $H^{\left(0\right)}$ corresponds to X the node feature matrix. The GCN is trained in series with the MME such that the weights of the MME are learnt jointly with the GCN. When training the network, nodes are randomly divided into training nodes and transductive test nodes. These transductive test nodes are not included in the loss computation, but they are still involved in the message passing algorithm (Hamilton 2020). This methodology means that although the labels are hidden, the graph does use test data to update its embedding representations. This methodology differs to classical supervised learning setting, in which the test data is removed from the model and is referred to as the semi-supervised training of GCN (Kipf and Welling 2017, Hamilton 2020).

### 4.5 Interpretability of MOGDx

Interpretability in biomedical applications is important to understand how specific features contribute to prediction so that therapeutic interventions or novel diagnoses can be well understood. MOGDx shows interpretability in a number of ways. First, through ablation experiments, we can identify which modalities are most predictive of the targeted outcome. Enrichment analysis is a well understood methodology to map selected genes to their biological and molecular pathways. Functional enrichment analysis was carried out using the Gene Set Enrichment Analysis algorithm by Shi and Walker (2007) in Python on the extracted features from the mRNA datasets. Similarly, enrichment analysis was carried out using the mCSEA (Martorell-Marugán *et al.* 2019) algorithm in R on the extracted CpG sites from the DNAm datasets. Results from these analyses were further compared to existing literature. Receiver Operator Characteristic and Area Under Curve plots are shown in Supplementary Figs S5–S7. Through these visualisations, we can assess which classes are most difficult to predict and obtain metrics for the overall accuracy of the model.

## Supplementary Material

btae523_Supplementary_Data

## Data Availability

All data are available to download from The Cancer Genome Atlas (TCGA)(https://www.cancer.gov/tcga). A script to download and obtain data exactly as it is presented is available from https://github.com/biomedicalinformaticsgroup/MOGDx.

## Code availability

All code is available for use from https://github.com/biomedicalinformaticsgroup/MOGDx or https://zenodo.org/doi/10.5281/zenodo.10545043. A tutorial is available at https://biomedicalinformaticsgroup.github.io/MOGDxBook/.
