# Supplementary material for: Multi-Omic Graph Diagnosis (MOGDx): a data integration tool to perform classification tasks for heterogeneous diseases
Source: Bioinformatics. 2024 Aug 23;40(9):btae523. doi: 10.1093/bioinformatics/btae523 (PMC11374023; doi:10.1093/bioinformatics/btae523)
Supplement: btae523_Supplementary_Data [file btae523_supplementary_data.pdf]

Multi-Omic Graph Diagnosis (MOGDx) : A data integration tool to perform classification tasks for heterogeneous diseases

1 Supplementary Figures

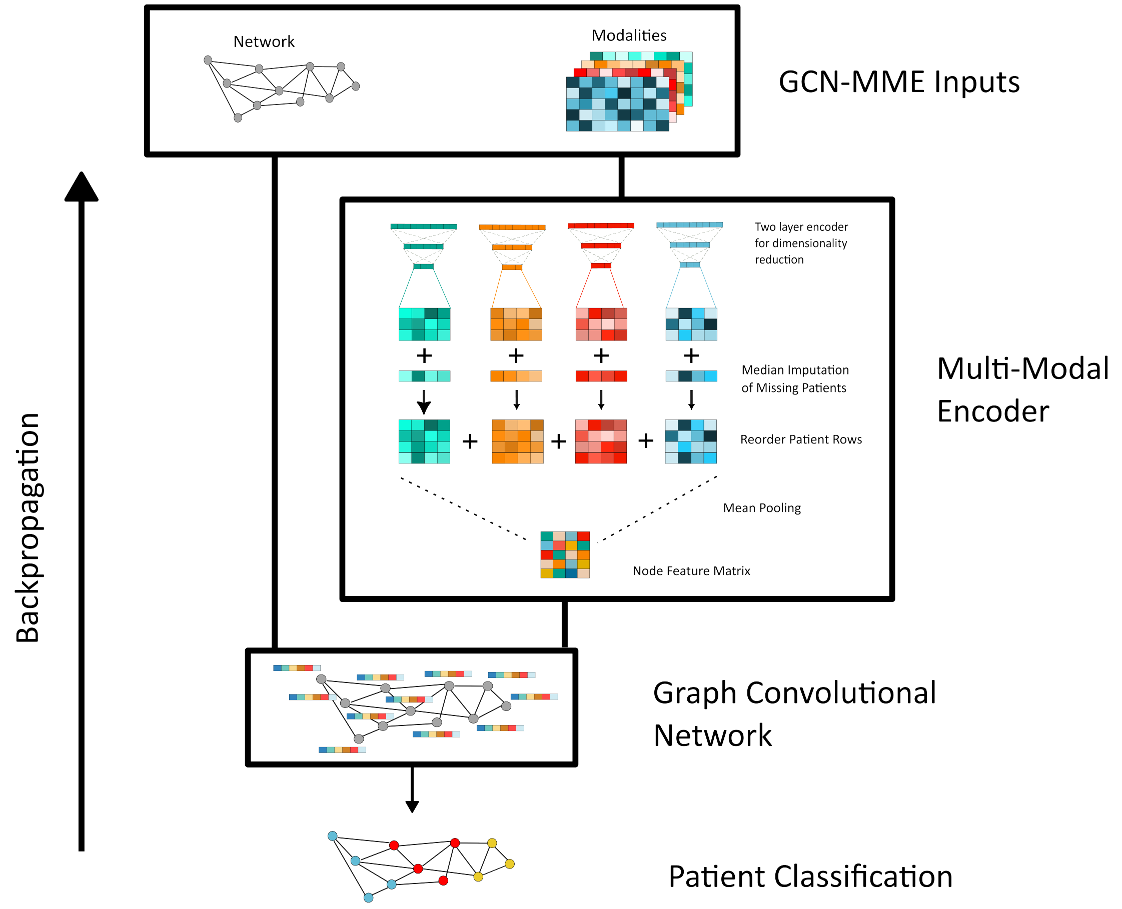

Figure 1: **Graph Convolutional Network - Multi-Modal Encoder Architecture (GCN-MME)** — The GCN-MME takes as input a fixed network and any number of modalities. The nodes in the network correspond to patients, and each patient is present in at least one modality. The modalities are encoded for dimensionality reduction using a two layer encoder. After the second layer, median imputation is performed to include patients missing from that modality but included in the network and at least one other modality. There is a shared latent embedding between the encoders, and the imputed second layers of each encoder are joined using mean pooling. This shared latent embedding forms the node features for the GCN. Patient classification is performed using the GCN with the loss back propagated through the entire GCN, thus, training each encoder in series with the GCN.

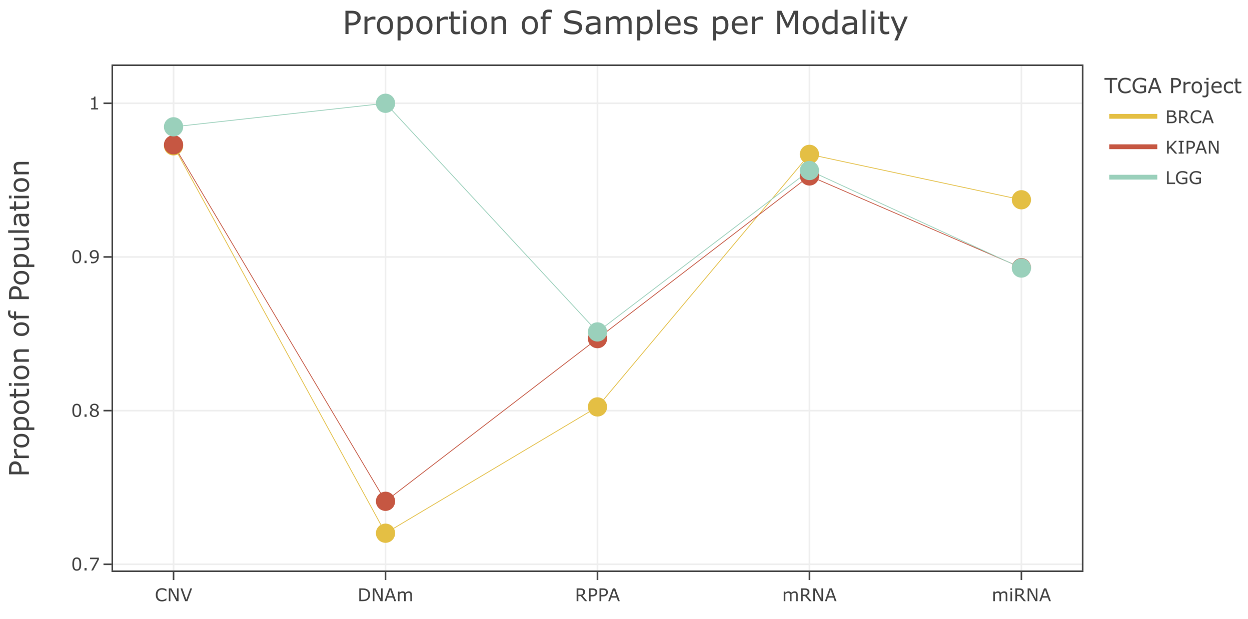

Figure 2: Number of Samples per Modality in TCGA Datasets

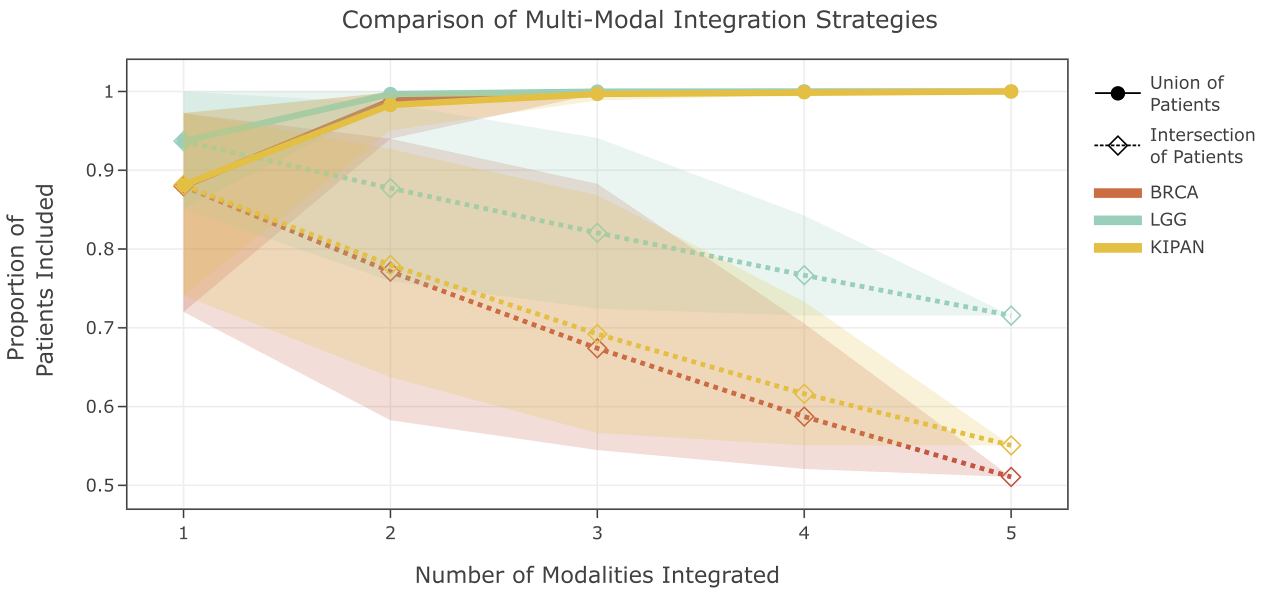

Figure 3: **Integration Strategy Comparison**— The effect of integrating the union of patients versus the intersection of patients on the TCGA dataset is shown. The lines show the mean percentage of patients used when integrating different numbers of modalities, and the fill represents the upper and lower bounds of patients included.

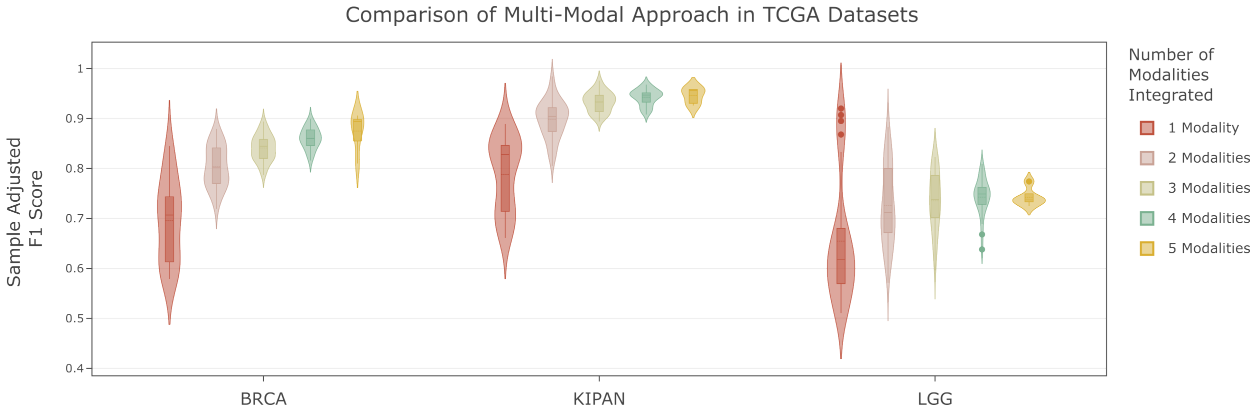

Figure 4: **MOGDx performance under different combinations of modalities** — F1 accuracy per number of integrated modalities across the three datasets. The distributions represent the F1 accuracy at each fold of each modality's model.

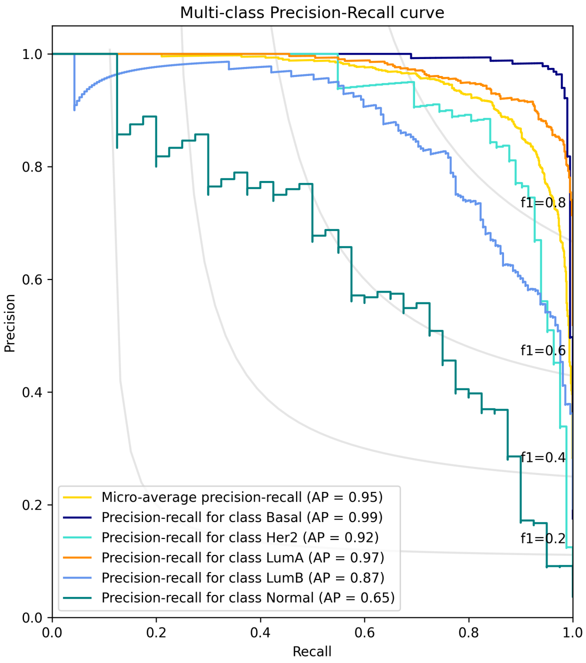

Figure 5: BRCA Precision Recall Curve

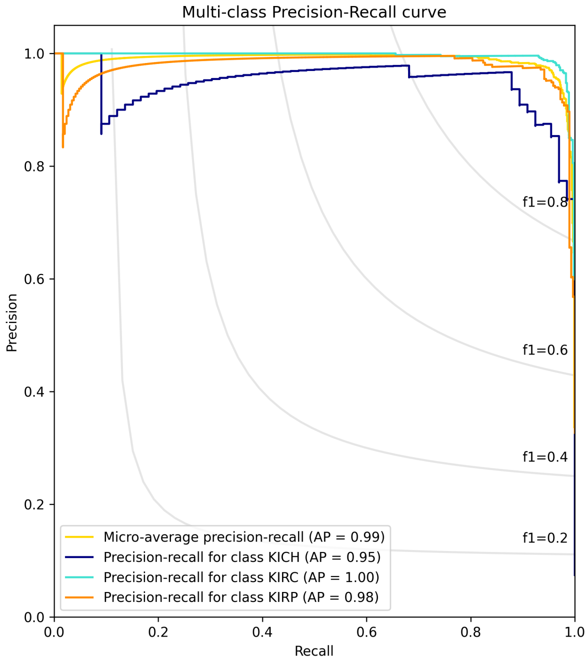

Figure 6: KIPAN Precision Recall Curve

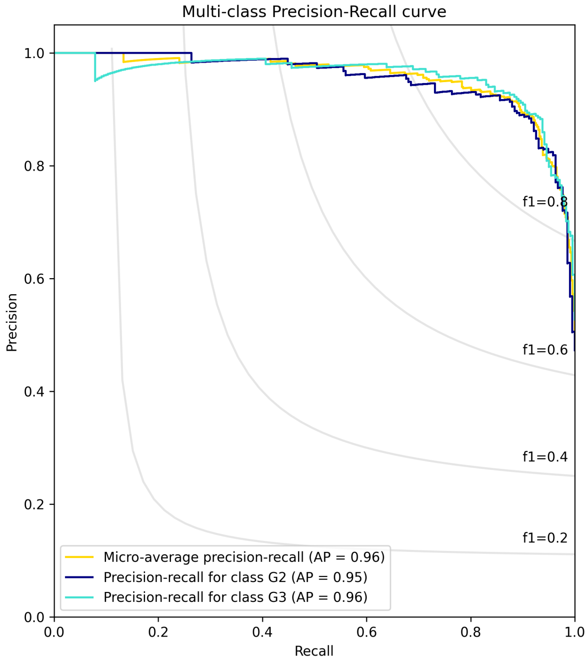

Figure 7: LGG Precision Recall Curve

2 Supplementary Tables

Table 1: Model Parameters for Optimal Performance on Each Dataset

Dataset Model	MME Modality Reduced Dimension					Shared Decoder Dimension	GCN Dimension
	mRNA	miRNA	DNAm	CNV	RPPA		
BRCA	32	X	32	16	16	32	32
LGG	X	X	32	X	X	32	32
KIPAN	X	16	32	16	16	64	128

Table 2: Summary of TCGA Dataset Features

Dataset	Modalities	Feature Counts		
		Raw	Processed	Extracted
BRCA	mRNA	60666	29995	2484
	miRNA	1881	1601	502
	DNAm	485577	200000	179
	RPPA	488	464	124
	CNV	60623	60265	474
LGG	mRNA	60660	22185	1000
	miRNA	1881	1515	200
	DNAm	485577	321999	318
	RPPA	487	457	65
	CNV	60623	60274	181
KIPAN	mRNA	60660	28212	1290
	miRNA	1881	1552	352
	DNAm	485577	310045	167
	RPPA	487	469	48
	CNV	60623	60274	157

Table 3: KIPAN: Optimal Modality Performance Summary. These accuracies are not weighted by the percentage of sample availability.

Project	Modality	Accuracy	F1
KIPAN	CNV	0.758 ± 0.02	0.741 ± 0.025
	DNAm	0.945 ± 0.024	0.931 ± 0.031
	RPPA	0.983 ± 0.018	0.977 ± 0.025
	mRNA	0.922 ± 0.017	0.905 ± 0.019
	miRNA	0.954 ± 0.014	0.943 ± 0.016
	CNV + RPPA	0.907 ± 0.026	0.891 ± 0.027
	DNAm + CNV	0.88 ± 0.028	0.855 ± 0.037
	DNAm + RPPA	0.966 ± 0.012	0.955 ± 0.018
	mRNA + CNV	0.899 ± 0.027	0.877 ± 0.035
	mRNA + DNAm	0.941 ± 0.009	0.924 ± 0.01
	mRNA + RPPA	0.954 ± 0.018	0.94 ± 0.023
	mRNA + miRNA	0.936 ± 0.017	0.921 ± 0.025
	miRNA + CNV	0.888 ± 0.017	0.87 ± 0.02
	miRNA + DNAm	0.955 ± 0.018	0.942 ± 0.023
	miRNA + RPPA	0.971 ± 0.019	0.964 ± 0.026
	DNAm + CNV + RPPA	0.948 ± 0.02	0.935 ± 0.022
	mRNA + CNV + RPPA	0.951 ± 0.01	0.938 ± 0.015
	mRNA + DNAm + CNV	0.939 ± 0.016	0.922 ± 0.019
	mRNA + DNAm + RPPA	0.957 ± 0.018	0.945 ± 0.024
	mRNA + miRNA + CNV	0.931 ± 0.025	0.915 ± 0.032
	mRNA + miRNA + DNAm	0.946 ± 0.024	0.931 ± 0.031
	mRNA + miRNA + RPPA	0.958 ± 0.011	0.945 ± 0.015
	miRNA + CNV + RPPA	0.949 ± 0.021	0.937 ± 0.024
	miRNA + DNAm + CNV	0.943 ± 0.013	0.931 ± 0.019
	miRNA + DNAm + RPPA	0.969 ± 0.018	0.961 ± 0.021
	mRNA + DNAm + CNV + RPPA	0.956 ± 0.018	0.945 ± 0.023
	mRNA + miRNA + CNV + RPPA	0.954 ± 0.015	0.942 ± 0.02
	mRNA + miRNA + DNAm + CNV	0.948 ± 0.01	0.935 ± 0.015
	mRNA + miRNA + DNAm + RPPA	0.959 ± 0.014	0.948 ± 0.019
	miRNA + DNAm + CNV + RPPA	0.958 ± 0.003	0.948 ± 0.004
	mRNA + miRNA + DNAm + CNV + RPPA	0.957 ± 0.012	0.946 ± 0.016

Table 4: LGG Optimal Modality Performance. These accuracies are not weighted by the percentage of sample availability.

Project	Modality	Accuracy	F1
LGG	CNV	0.656 ± 0.046	0.641 ± 0.042
	DNAm	0.904 ± 0.014	0.887 ± 0.016
	RPPA	0.65 ± 0.029	0.636 ± 0.033
	mRNA	0.689 ± 0.036	0.663 ± 0.034
	miRNA	0.677 ± 0.063	0.652 ± 0.055
	CNV + RPPA	0.727 ± 0.04	0.7 ± 0.035
	DNAm + CNV	0.849 ± 0.025	0.824 ± 0.027
	DNAm + RPPA	0.849 ± 0.032	0.827 ± 0.036
	mRNA + CNV	0.724 ± 0.024	0.692 ± 0.024
	mRNA + DNAm	0.768 ± 0.043	0.739 ± 0.045
	mRNA + RPPA	0.716 ± 0.04	0.692 ± 0.04
	mRNA + miRNA	0.698 ± 0.052	0.677 ± 0.053
	miRNA + CNV	0.684 ± 0.052	0.659 ± 0.048
	miRNA + DNAm	0.845 ± 0.029	0.822 ± 0.03
	miRNA + RPPA	0.669 ± 0.033	0.642 ± 0.031
	DNAm + CNV + RPPA	0.818 ± 0.013	0.791 ± 0.012
	mRNA + CNV + RPPA	0.724 ± 0.032	0.703 ± 0.027
	mRNA + DNAm + CNV	0.796 ± 0.029	0.768 ± 0.032
	mRNA + DNAm + RPPA	0.768 ± 0.009	0.74 ± 0.011
	mRNA + miRNA + CNV	0.722 ± 0.042	0.7 ± 0.04
	mRNA + miRNA + DNAm	0.746 ± 0.045	0.717 ± 0.044
	mRNA + miRNA + RPPA	0.697 ± 0.029	0.68 ± 0.027
	miRNA + CNV + RPPA	0.696 ± 0.059	0.671 ± 0.044
	miRNA + DNAm + CNV	0.825 ± 0.021	0.798 ± 0.024
	miRNA + DNAm + RPPA	0.81 ± 0.034	0.781 ± 0.037
	mRNA + DNAm + CNV + RPPA	0.79 ± 0.024	0.762 ± 0.023
	mRNA + miRNA + CNV + RPPA	0.704 ± 0.047	0.689 ± 0.039
	mRNA + miRNA + DNAm + CNV	0.785 ± 0.036	0.76 ± 0.034
	mRNA + miRNA + DNAm + RPPA	0.777 ± 0.023	0.746 ± 0.024
	miRNA + DNAm + CNV + RPPA	0.785 ± 0.021	0.756 ± 0.022
	mRNA + miRNA + DNAm + CNV + RPPA	0.77 ± 0.021	0.743 ± 0.019

Table 5: BRCA Optimal Modality Performance. These accuracies are not weighted by the percentage of sample availability.

Project	Modality	Accuracy	F1
	CNV	0.707 ± 0.029	0.729 ± 0.03
	DNAm	0.839 ± 0.017	0.826 ± 0.019
	RPPA	0.8 ± 0.018	0.776 ± 0.02
	mRNA	0.865 ± 0.021	0.84 ± 0.025
	miRNA	0.795 ± 0.028	0.757 ± 0.038
	CNV + RPPA	0.803 ± 0.015	0.777 ± 0.021
	DNAm + CNV	0.778 ± 0.032	0.765 ± 0.031
	DNAm + RPPA	0.829 ± 0.028	0.815 ± 0.027
	mRNA + CNV	0.865 ± 0.01	0.839 ± 0.012
	mRNA + DNAm	0.879 ± 0.018	0.856 ± 0.021
	mRNA + RPPA	0.869 ± 0.015	0.845 ± 0.017
	mRNA + miRNA	0.862 ± 0.025	0.835 ± 0.03
	miRNA + CNV	0.783 ± 0.014	0.763 ± 0.02
	miRNA + DNAm	0.82 ± 0.01	0.803 ± 0.007
	miRNA + RPPA	0.812 ± 0.036	0.795 ± 0.039
BRCA	DNAm + CNV + RPPA	0.843 ± 0.016	0.825 ± 0.02
	mRNA + CNV + RPPA	0.873 ± 0.014	0.852 ± 0.015
	mRNA + DNAm + CNV	0.878 ± 0.025	0.858 ± 0.03
	mRNA + DNAm + RPPA	0.881 ± 0.037	0.862 ± 0.041
	mRNA + miRNA + CNV	0.866 ± 0.014	0.847 ± 0.017
	mRNA + miRNA + DNAm	0.877 ± 0.013	0.858 ± 0.016
	mRNA + miRNA + RPPA	0.872 ± 0.021	0.855 ± 0.013
	miRNA + CNV + RPPA	0.824 ± 0.015	0.812 ± 0.012
	miRNA + DNAm + CNV	0.83 ± 0.016	0.808 ± 0.019
	miRNA + DNAm + RPPA	0.852 ± 0.028	0.834 ± 0.024
	mRNA + DNAm + CNV + RPPA	0.884 ± 0.012	0.864 ± 0.012
	mRNA + miRNA + CNV + RPPA	0.879 ± 0.013	0.858 ± 0.016
	mRNA + miRNA + DNAm + CNV	0.885 ± 0.017	0.863 ± 0.022
	mRNA + miRNA + DNAm + RPPA	0.892 ± 0.008	0.873 ± 0.011
	miRNA + DNAm + CNV + RPPA	0.849 ± 0.007	0.828 ± 0.009
	mRNA + miRNA + DNAm + CNV + RPPA	0.893 ± 0.035	0.875 ± 0.039

Table 6: BRCA Component Performance Fold Summary

	BRCA					
	PSN + MME		PSN		MME	
	Accuracy	F1	Accuracy	F1	Accuracy	F1
Fold 1	0.903	0.884	0.871	0.839	0.857	0.833
Fold 2	0.894	0.870	0.88	0.85	0.829	0.795
Fold 3	0.871	0.857	0.899	0.885	0.834	0.816
Fold 4	0.889	0.869	0.907	0.886	0.843	0.82
Fold 5	0.907	0.890	0.861	0.83	0.843	0.821

Table 7: LGG Component Performance Fold Summary

	LGG					
	PSN + MME		PSN		MME	
	Accuracy	F1	Accuracy	F1	Accuracy	F1
Fold 1	0.924	0.907	0.902	0.882	0.721	0.718
Fold 2	0.913	0.895	0.924	0.909	0.685	0.662
Fold 3	0.857	0.833	0.912	0.896	0.703	0.674
Fold 4	0.934	0.92	0.879	0.856	0.648	0.672
Fold 5	0.89	0.868	0.901	0.883	0.681	0.652

Table 8: KIPAN Component Performance Fold Summary

	KIPAN					
	PSN + MME		PSN		MME	
	Accuracy	F1	Accuracy	F1	Accuracy	F1
Fold 1	0.955	0.945	0.966	0.956	0.972	0.962
Fold 2	0.961	0.949	0.966	0.956	0.972	0.964
Fold 3	0.961	0.951	0.978	0.971	0.961	0.954
Fold 4	0.96	0.951	0.955	0.943	0.994	0.993
Fold 5	0.955	0.942	0.966	0.955	0.977	0.971

Table 9: TCGA DNAm Over Representation Analysis Results

Dataset	Gene set	Term	Overlap	P-value	Adjusted P-value	Odds Ratio
BRCA	MSigDB Hallmark 2020	Estrogen Response Early	100/200	1.02445e-10	5.12225e-09	2.53256
		Epithelial Mesenchymal Transition	95/200	8.87321e-09	2.2183e-07	2.28852
		Myogenesis	91/200	2.20846e-07	3.68076e-06	2.10961
	KEGG 2021 Human	Axon guidance	103/182	1.7986e-15	5.71956e-13	3.3086
		Proteoglycans in cancer	108/205	2.52725e-13	4.01832e-11	2.82438
		Pathways in cancer	227/531	9.28599e-13	7.96066e-11	1.90712
KIPAN	MSigDB Hallmark 2020	Estrogen Response Early	106/200	1.19312e-11	5.96561e-10	2.63818
		Myogenesis	98/200	1.62511e-08	4.06277e-07	2.24345
		Epithelial Mesenchymal Transition	93/200	7.80117e-07	9.75147e-06	2.02706
	KEGG 2021 Human	Axon guidance	110/182	2.22215e-17	7.04421e-15	3.58235
		Proteoglycans in cancer	113/205	7.72699e-14	1.22473e-11	2.87735
		Human papillomavirus infection	163/331	1.92237e-13	2.0313e-11	2.2797
LGG	MSigDB Hallmark 2020	Estrogen Response Early	108/200	2.62026e-11	1.31013e-09	2.58899
		Epithelial Mesenchymal Transition	104/200	1.05084e-09	2.62711e-08	2.38698
		Apical Junction	98/200	1.47716e-07	2.46193e-06	2.11397
	KEGG 2021 Human	Axon guidance	113/182	1.28249e-17	4.0783e-15	3.62084
		Focal adhesion	121/201	3.05032e-17	4.85001e-15	3.34571
		Proteoglycans in cancer	122/205	8.00606e-17	8.48642e-15	3.25124

Table 10: TCGA mRNA Gene Set Enrichment Analysis Results

Dataset	Gene set	Term	ES	NES	NOM p-val	FDR q-val	Tag %	Gene %
BRCA	KEGG 2021 Human	Pentose and glucuronate interconversions	-0.774355	-2.29015	0	0	10/16	9.58%
		Cell cycle	0.573984	2.27577	0	0	48/108	16.26%
		Homologous recombination	-0.649829	-2.39384	0	0	13/25	14.04%
	MSigDB Hallmark 2020	G2-M Checkpoint	0.576235	2.46117	0	0	102/188	23.18%
		Androgen Response	-0.499048	-2.03848	0	0.00577345	37/68	25.03%
		Myc Targets V1	0.446703	1.88204	0	0.00801285	129/175	46.57%
LGG	KEGG 2021 Human	Nicotine addiction	0.80602	3.10307	0	0	15/18	11.28%
		Systemic lupus erythematosus	-0.641083	-2.29704	0	0	35/52	20.32%
		Type I diabetes mellitus	-0.737799	-2.2463	0	0	19/24	15.40%
	MSigDB Hallmark 2020	G2-M Checkpoint	-0.672679	-2.6506	0	0	53/126	8.53%
		E2F Targets	-0.641025	-2.52297	0	0	66/137	15.34%
		Epithelial Mesenchymal Transition	-0.621457	-2.45649	0	0	94/144	23.64%
KIPAN	KEGG 2021 Human	Oxidative phosphorylation	0.623763	3.14138	0	0	87/112	24.92%
		Pentose and glucuronate interconversions	-0.668482	-2.11695	0	0	11/24	10.08%
		Intestinal immune network for IgA production	-0.624027	-2.15917	0	0	31/40	27.41%
	MSigDB Hallmark 2020	Oxidative Phosphorylation	0.604961	3.25895	0	0	145/196	27.66%
		Allograft Rejection	-0.562653	-2.43625	0	0	118/178	27.41%
		Epithelial Mesenchymal Transition	-0.527188	-2.26799	0	0	111/166	30.88%
